# The Australian longitudinal study of health and relationships

**DOI:** 10.1186/1471-2458-7-139

**Published:** 2007-07-04

**Authors:** Anthony MA Smith, Marian K Pitts, Julia M Shelley, Juliet Richters, Jason Ferris

**Affiliations:** 1 Australian Research Centre in Sex, Health & Society, La Trobe University, 215 Franklin St, Victoria 3000, Australia; 2 National Centre in HIV Social Research, University of New South Wales, Sydney, New South Wales 2052, Australia

## Abstract

**Background:**

Ensuring the sexual and reproductive health of the population is essential for the wellbeing of a nation. At least three aspects of sexual and reproductive health are among the key policy issues for present Australian governments: maintaining and increasing the birth rate; reducing the abortion rate; and preventing and controlling Chlamydia infections.

The overall aim of the Australian Longitudinal Study of Health and Relationships is to document the natural history of the sexual and reproductive health of the Australian adult population.

**Methods/design:**

A nationally representative sample of Australian adults 16–64 years of age was selected in a two-phase process in 2004–2005. Eligible households were identified through random digit dialling. We used separate sampling frames for men and women; where there was more than one eligible person in a household the participant was selected randomly. Participants completed a computer-assisted telephone interview that typically took approximately 25 minutes to complete. The response rate was 56%. A total of 8,656 people were interviewed, of whom 95% (8243) agreed to be contacted again 12 months later. Of those, approximately 82% have been re-contacted and re-interviewed in 2006–07 (Wave Two), with 99% of those agreeing to be contacted again for Wave Three.

**Discussion:**

ALSHR represents a significant advance for research on the linked topics of sexual and reproductive health. Its strengths include the large sample size, the inclusion of men as well as women, and the wide age range of the participants.

## Background

*"Sexual and reproductive health is fundamental to the social and economic development of communities and nations, and a key component of an equitable society"*.

*"Sexual health and reproductive health overlap and, in addition to supporting normal physiological functions such as pregnancy and childbirth, aim to reduce adverse outcomes of sexual activity and reproduction. They are also about enabling people of all ages, including adolescents and those older than the reproductive years, to have safe and satisfying sexual relationships by tackling obstacles such as gender discrimination, inequalities in access to health services, restrictive laws, sexual coercion, exploitation, and gender-based violence." *[1]

As presented so clearly in the special *Lancet *series on sexual and reproductive health in November/December 2006 [1], ensuring the sexual and reproductive health of the Australian population is essential for the wellbeing of the nation. As well as their overall importance, at least three aspects of sexual and reproductive health are among the key policy issues for present Australian governments: maintaining and increasing the birth rate; reducing the abortion rate; and preventing and controlling Chlamydia infections.

Yet sexual and reproductive health, as a broad and interrelated set of health outcomes, has been the subject of relatively little public health examination. Despite calls for a national sexual health strategy [2], and the existence of the National Indigenous Australians Sexual Health Strategy [3], the National HIV/AIDS Strategy [4] and the National Sexually Transmitted Infection (STI) Strategy [5], the area lacks a comprehensive policy framework. Thus, for example, safe sex practices and contraceptive practices are inextricably linked yet the competing policy imperatives are rarely identified. There are currently no data to demonstrate whether, for example, promoting condom use to adolescents for HIV/STI prevention may have unintended effects such as discouraging the use of more effective contraceptive methods.

Analysis of general practice activity in 2005–06 indicated that one in 15 GP consultations concerned sexual and reproductive health. As a percentage of total reasons for encounters, sexual and reproductive health matters comprised 6.5%. In comparison, cardiovascular reasons comprised 7.2% and digestive reasons 6.6% of total reasons for consultation. [6]

STIs are common in Australia and are responsible for a significant amount of long-term morbidity. Chlamydia, for example, is now the most common notifiable infection in Australia with 43,681 notifications in 2006 and is a significant cause of infertility at a time when Australia's population growth is at its lowest. [5]

There have been five major longitudinal studies in the US that have begun to map similar territory. They are: the Wisconsin Longitudinal Study (WLS) [7], The Health and Retirement Survey (HRS) [8], The National Survey of Families and Households (NSFH) [9], American Changing Lives (ACL) [10] and Changing Lives of Older Couples (CLOC) [11]. Of these only WLS and HRS are ongoing.

Table 1 indicates the significant gaps in information emanating from such studies and compares them with the Australian Longitudinal Study of Health and Relationships (ALSHR).

**Table 1:** Sexual and reproductive health data from longitudinal studies

**Variable**	**HRS**	**WLS**	**NSFH**	**ACL**	**CLOC**	**ALSHR**
Fertility and reproductive history	Excellent	Excellent	Excellent	Good	Excellent	Excellent
Sexuality and sexual relationships	None	None	Good	None	None	Excellent
Women's reproductive health	Good	None	Some	None	None	Good
Men's reproductive health	None	None	Some	None	None	Good

There are other longitudinal studies, but all have focused either on adolescents (National Longitudinal Study of Adolescent Health (USA), Christchurch Health and Development Study (NZ), National Adolescent Males and Youth Risk Behavior Survey (USA), National Longitudinal Surveys of Youth '79 and '97 (USA); or only on women (Australian Women's Health Study, Melbourne Women's Midlife Health Study, Iowa Women's Health Study (USA), Reproductive Risk Factors for Incontinence Study at Kaiser (USA). Men are seriously under-studied and the complex relational elements of sexual and reproductive health decisions and outcomes are barely touched upon.

The overall aim of ALSHR is to document the natural history of the sexual and reproductive health of the Australian adult population.

## Methods/design

ALSHR was funded by the NHMRC for the period 2003–2007 to conduct computer-assisted telephone interviews annually with a large broadly representative national sample of Australians aged 16–64 years at recruitment.

Eligible households were identified through random digit dialling, as in the Australian Study of Health and Relationships, on which this study is partly based. [12] We used separate sampling frames for men and women; where there was more than one eligible person in a household the participant was selected randomly. Participants completed a computer-assisted telephone interview that typically took approximately 25 minutes to complete. The interview was available only in English and hence people with insufficient proficiency in English (about 1%) could not participate. All participants gave verbal informed consent.

This research was approved by the Human Ethics Committees of La Trobe University, the University of New South Wales and Deakin University.

The response rate was 56%. Initially a total of 8,656 people were interviewed, of whom 95% (8243) agreed to be contacted again 12 months later for re-interview. Of those, approximately 82% have been re-contacted and re-interviewed in 2006–07 (Wave Two), with 99% agreeing to be contacted again for Wave Three.

We stopped recruiting in Wave One earlier than anticipated because we achieved our target of enrolling participants for Wave Two. Over 96% of respondents to Wave One agreed to participate in Wave Two. This high participation rate for the cohort can be attributed to the professionalism and high standards of the interviewers. High retention rates thereafter are due to (1) the use of a tracking call after six months to those believed most likely to be lost (i.e. people under 36 who had been at their current home for less than three years); and (2) requesting multiple contact details from cohort participants (phone numbers, email addresses, and contact details of friends in case we lost contact with the cohort member). This leads us to anticipate a further retention rate of 95% although in practice this is a worst-case estimate. As in all cohorts, the greatest loss to follow-up is expected to be that between Waves One and Two. A loss to follow-up of 18% compares extremely well to that observed for Women's Health Australia youngest cohort (31% of women aged 18–23) but is not as good as the loss to follow up of the mid-aged (45–50 years) and older (70–75 years) cohorts (10% for both).

In the first year, the interview included: sexual history; sexual activity in the last month and last year; experience of sexual problems; contraceptive practices; experience of pregnancy and pregnancy outcomes (live-birth, termination, miscarriage or still-birth); experience of tubal ligation, hysterectomy or vasectomy; physical and emotional satisfaction with sexual relationships; attitudes to issues such as termination and homosexuality; and, the use of health services relevant to sexual and reproductive health; demographic information (age, gender, ethnicity; household size and composition; legal marital status and relationship status); relevant medical conditions and health status (diagnoses of diabetes, hypertension, disability; use of relevant medication such as antihypertensives or antidepressants); emotional and mental well-being; tobacco, alcohol and other drug use.

During the first interview, the contact details of three people likely to know the whereabouts of the participant were obtained. These were verified on each subsequent contact.

During follow-up, a slightly shorter version of the questionnaire was used. It collected identical information to the intake interview except that it will assess relevant outcomes only in the period since the last interview.

The study research questions, relevant sections of the interview and the benefit to be derived from the study appear in Table 2.

**Table 2:** Study research questions, interview sections and derived benefit

**Question**	**Section(s)**	**Benefit**
What is the relationship between the age of sexual debut, patterns of sexual activity, and subsequent sexual functioning and reproductive health including diagnosis with STI's?	Sexual debut – first sex Sexual history Sexual function (noted by dysfunction) Reproductive history and reproductive future STI's	To describe patterns of sexual behaviour and thus better direct public education programs to reduce STI To better understand the impact of early/delayed sexual activity on reproductive health
What are the sociodemographic, sexual and reproductive predictors of contraceptive choice and change?	Demographics Contraceptive history/use Reproductive history/use Sexual history/current Last sex	To follow the change in contraception (and use) due to sociodemographic markers and sexual history. To determine the impact of this change on reproductive outcomes and birth rates
What is the nature and magnitude of the relationship between experiencing sexual coercion and subsequent sexual functioning and reproductive health?	Sexual coercion Sexual history/future Reproductive history/future	To identify the ongoing impact of sexual coercion (to monitor changes – on a yearly basis rather than recall over time) To provide insight into the long term effects of sexual coercion
What are the sociodemographic, sexual and reproductive predictors of tubal ligation, vasectomy and hysterectomy?	Demographics Contraceptive history/use Reproductive history/use Sexual activity history/current	To identify markers for (semi) permanent surgery as a method of reproductive control and sexual behaviour
What is the impact of tubal ligation, vasectomy, and hysterectomy on subsequent sexual functioning?	Sexual activity current/future Sexual function/dysfunction General health	As the study is longitudinal we can determine the impact of reproductive surgeries on subsequent sexual functioning – monitoring changes yearly as they occur
What are the sociodemographic, sexual and reproductive predictors of pregnancy and pregnancy outcomes, particularly abortion and miscarriage?	Demographics Contraceptive history/use Reproductive history/use Enumeration of pregnancies Sexual activity history/current Expectancies of future pregnancies Last sex	Pregnancy/birth history and reproductive history can provide insight to abortion and miscarriage outcomes
What are the impacts of spontaneous and induced pregnancy losses on subsequent sexual functioning and reproductive outcomes including contraceptive choice?	Contraceptive history/use Reproductive history/use Sexual activity history/current Sexual function/dysfunction General health	Pregnancy loss can have an impact of mental and general health and sexual functioning. Monitoring this can provide insight into the ongoing effects of pregnancy loss
What is the relationship between sexual functioning, reproductive health and satisfaction with the physical and emotional aspects of people's intimate relationships and subsequent relationship breakdown?	Sexual satisfaction/expectation Physical satisfaction/expectation Emotional satisfaction/expectation Reproductive history/use Sexual activity history/current Relationship enumeration	To provide a template of the interplay between satisfaction, expectation and relationship history. Monitor changes against the marker of expectations
What is the impact of divorce or other relationship breakdown on subsequent sexual functioning and reproductive health?	Marriage/divorce Duration of relationships Sexual function/dysfunction Sexual activity history/current Reproductive history Relationship enumeration	To measure markers of relationship breakdown against future relationships and sexual functioning To monitor the dynamic changes in relationships with a focus on sexual functioning and reproductive health

## Discussion

ALSHR represents a significant advance for research on the linked topics of sexual and reproductive health. Its strengths include the large sample size, the inclusion of men as well as women, and the wide age range of the participants.

Concern is often expressed that reliable results cannot be achieved in telephone surveys on sensitive topics such as sex and reproduction (e.g. abortion) – in short, that people do not tell the truth. [12] However, our experience on previous surveys [12,13] shows that with good questionnaire design, results as good as or better than for any other health topics can be achieved in sex surveys. There is little evidence of bias due to social desirability except for the tendency of men to report more sexual partners and more sexual practices than women. A complex survey such as ALSHR with many interconnected questions requires sustained consistency in responses; most people cannot achieve this except by (more or less) telling the truth. Although some stigmatised events may be suppressed in respondents' memories and not reported – for example surrender of a child for adoption in the 1960s – for many the telephone survey is the first opportunity to tell the truth in a non-judgmental and anonymous setting. Our confidence in the accuracy of ALSHR's data is confirmed by the correspondence of our findings with external sources and with the earlier surveys. [12,13]

## Competing interests

The author(s) declare that they have no competing interests.

## Authors' contributions

AS led study design and writing. All authors contributed to study design and drafting the manuscript. All read and approved the final manuscript.

## Pre-publication history

The pre-publication history for this paper can be accessed here:


